# Factors associated with modern contraceptive use among women with no fertility intention in sub-Saharan Africa: evidence from cross-sectional surveys of 29 countries

**DOI:** 10.1186/s40834-021-00165-6

**Published:** 2021-08-01

**Authors:** Bright Opoku Ahinkorah, Eugene Budu, Richard Gyan Aboagye, Ebenezer Agbaglo, Francis Arthur-Holmes, Collins Adu, Anita Gracious Archer, Yaa Boahemaa Gyasi Aderoju, Abdul-Aziz Seidu

**Affiliations:** 1 School of Public Health, Faculty of Health, University of Technology Sydney, Sydney, Australia; 2 Department of Population and Health, University of Cape Coast, Cape Coast, Ghana; 3 School of Public Health, University of Health and Allied Sciences, Ho, Ghana; 4 Department of English, University of Cape Coast, Cape Coast, Ghana; 5 Department of Sociology and Social Policy, Lingnan University, 8 Castle Peak Road, Tuen Mun, Hong Kong; 6 Department of Health Promotion, Education and Disability Studies, Kwame Nkrumah University of Science and Technology, Kumasi, Ghana; 7 School of Nursing and Midwifery, University of Health and Allied Sciences, Ho, Ghana; 8 Department of Adult Health Nursing, School of Nursing and Midwifery, University of Cape Coast, Cape Coast, Ghana; 9 College of Public Health, Medical and Veterinary Services, James Cook University, Townsville, Australia

**Keywords:** Fertility intention, modern contraceptives, sub-Saharan Africa, Women, Women’s health

## Abstract

**Background:**

In sub-Saharan Africa, the majority of women of reproductive age who want to avoid pregnancy do not use any method of contraception. This study sought to determine the factors associated with  modern contraceptive use among women with no fertility intention in sub-Saharan Africa.

**Methods:**

This study used data from the Demographic and Health Surveys of 29 countries in sub-Saharan Africa. A total of 87,554 women aged 15–49 with no fertility intention and who had completed information on all the variables of interest were considered in this study. Using a multilevel logistic regression analysis, four models were used to examine the individual and contextual factors associated with modern contraceptive use. The results were presented as adjusted odds ratios (aOR), with their respective confidence intervals (CIs). Statistical significance was set at p< 0.05.

**Results:**

The prevalence of modern contraceptive use was 29.6%. With the individual-level factors, women aged 45–49 had lower odds of using modern contraceptives (aOR = 0.33, 95% CI = 0.28, 0.39). Women who had their first sex at age 15–19 (aOR = 1.12, 95% CI = 1.07, 1.17), those with higher education (aOR = 1.93, 95% CI = 1.75, 2.13), and women who were exposed to newspaper (aOR = 1.15, 95% CI = 1.10, 1.20) and radio (aOR = 1.21, 95% CI = 1.17, 1.26) had higher odds of modern contraceptive use. In terms of the contextual factors, women living in urban areas (aOR = 1.06, 95% CI = 1.02, 1.11), women in the richest wealth quintile (aOR = 1.55, 95% CI = 1.43, 1.67), and those in communities with medium literacy level (aOR = 1.11, 95% CI = 1.06, 1.16) and medium community socio-economic status (aOR = 1.17, 95% CI = 1.10, 1.23) had higher odds of modern contraceptive use. Across the geographic regions in sub-Saharan Africa, women in Southern Africa had higher odds of modern contraceptive use (aOR = 5.29, 95% CI = 4.86, 5.76).

**Conclusion:**

There is a relatively low prevalence of modern contraceptive use among women with no fertility intention in sub-Saharan Africa, with cross-country variations. Women’s age, age at first sex, level of education, mass media exposure, place of residence, community literacy level and community socio-economic status were found to be associated with modern contraceptive use. It is, therefore, important for policy makers  to consider these factors when designing and implementing programmes or policies  to increase contraceptive use among women who have no intention to give birth. Also, policymakers and other key stakeholders should intensify mass education programmes to address disparities in modern contraceptive use among women.

## Background

To achieve target 3.7 of the Sustainable Development Goal (SDG) 3 which emphasizes that by 2030 the world should ensure universal access to sexual and reproductive healthcare services, utilization of modern contraceptive is key [1]. Report indicates that modern contraception prevented about 308 million unintended pregnancies in 2017 [2]. An additional 67 million unintended pregnancies could be averted if the needs for modern contraceptives are met [2]. Provision of modern contraception to women who want to avoid pregnancy could cause a reduction in maternal deaths from 308,000 to 84,000 and newborn mortality from 2.7 million to 538,000 per year respectively [3]. These reductions in unintended pregnancies, and maternal and newborn mortalities can lead to the attainment of the target 3.1 and 3.2 of the SDG 3 which seek to reduce the global maternal mortality ratio to less than 70 per 100,000 live births and end all preventable deaths under 5 years of age by 2030 respectively [1].

Currently, about 1.9 billion women of reproductive age are living in the world. Of this number, 1.1 billion women need family planning [2, 4].  Only 842 million used modern contraceptives in 2019 [2, 4]. However, about 190 million women of reproductive age worldwide who want to avoid pregnancy do not use any contraceptive method, up from 156 million in 2000 [5]. In low-and middle-income countries, 214 million women who wanted to avoid pregnancy in 2019 were not using any method of contraception [2].

In sub-Saharan Africa (SSA), the majority of women of reproductive age who want to avoid pregnancy do not use any method of contraception [6]. Data from the United Nations Department of Economic and Social Affairs and Population Division [5] show that more than 20% of unmet need for family planning were in 15 countries in SSA. A study also reveals that 51 million women of reproductive age had an unmet need for modern contraceptive methods [4]. However, disparities exist in modern contraceptive utilization among women of reproductive age in SSA.

Evidence suggests that the prevalence of modern contraceptive usage among women of reproductive age in 2019 was high in both Estwatini and Namibia (both reporting 52%) and low in Sudan (4%) [5]. Among Ethiopian women, the prevalence of contraceptive use was 51.1% [7]. Also, Gebrecherkos et al. [8] found a 41.8% prevalence of unmet need for modern contraception of which 31.8 and 10% represented the unmet need for spacing and limiting of birth respectively among women of reproductive age in Ethiopia. In Ghana, Wulifa et al. [9] found that 14.98% of the women had an unmet need for modern contraceptives. In Botswana, Letamo and Navaneetham [10] found the unmet need for family planning was reported to be 9.6% among married women, 6.7% for spacing, and 2.9% for limiting. Despite the existing data on unmet needs for family planning, there is still  a paucity of data on the magnitude of modern contraception among fecund women or women of reproductive age who do not have the desire to conceive a child in SSA.

Previous studies have suggested that cultural and religious myths and misconceptions undermine modern contraception [9, 11]. Other studies have highlighted that couple related factors (such as partner discussion, approval, and spousal decision making) [10, 12, 13], sociodemographic characteristics (age, educational status, religion, etc.) [10, 14], parity, exposure to mass media [14], knowledge on modern contraceptive methods [15], and fear of side effects [16] are associated with the utilization of modern contraceptives.

Although studies have recognized the importance of modern methods of contraception, few studies have been conducted to determine the factors associated with its utilization among women who have no intention to give birth to another child. This study sought to determine the factors associated with modern contraceptive use among women with no fertility intention in SSA. The findings from this study will provide evidence to inform decision-makers and stakeholders involved in family planning to improve women's access to sexual and reproductive health services, specifically, modern contraceptives in SSA.

## Methods

### Study design

Data for this study were obtained from the Demographic and Health Surveys (DHS) of 29 countries in SSA. For the purpose of the study, the women’s recode files, which contain data on women aged 15–49 were used. The DHS is a nationally representative survey that is conducted in over 85 low- and middle-income countries and focuses on important men, maternal, and child health markers such as contraceptive use [17]. The survey employs a two-stage stratified sampling technique, which makes the data nationally representative. The study by Aliaga and Ruilin [18] provides details of the sampling process. A total of 87,544 women aged 15–49 who had no intention to give birth and had complete information on all the variables of interest in this study were considered. In this study, women with no fertility intention were those who responded “want no more,” to the DHS question “would you like to have a (another) child with your husband/partner, or would you prefer not to have any more children with him?” We relied on the ‘Strengthening the Reporting of Observational Studies in Epidemiology’ (STROBE) statement in writing the manuscript [19]. The dataset is freely available for download at: https://dhsprogram.com/data/available-datasets.cfm.

### Variables studied

#### Outcome variable

The outcome variable for the study was current use of modern contraceptives among women with no fertility intention. We focused on women with no fertility intention as we expected that those category of women would be more likely to use contraceptives to prevent pregnancy. Hence, understanding the use of modern contraceptives among this cohort of women and the factors that drive them to use modern contraceptives is very crucial to reducing unwanted pregnancies and abortions. The variable, modern contraceptives, was derived from a question that elicited the types of contraceptives women with no fertility intention were using during the survey. Responses to this question were coded as “no method”, “folkloric method”, “traditional method” and “modern method”. The modern methods included female sterilization, male sterilization, intrauterine device (IUD), injectables, and implants (Norplant). The modern methods also included contraceptive pill, condoms, emergency contraception, standard day method (SDM), vaginal methods (foam, jelly, suppository), and lactational amenorrhea method (LAM). Country-specific modern methods and other modern contraceptive methods (including cervical cap, contraceptive sponge, and others)which were mentioned by respondents were also regarded as modern contraceptives. Periodic abstinence (rhythm, calendar method), withdrawal (coitus interruptus) and country-specific traditional methods which are proven effectivewere considered as traditional methods. Locally described methods and spiritual methods (such as herbs, amulets, gris-gris, etc.) which are effective but not proven were the folkloric methods [17, 20, 21]. The existing DHS variable on contraceptive use did not include women who were pregnant, those who were infecund, and those who had never had sex. In this study, women using modern methods were coded as ‘1’ while those who were not using any methods, those using traditional methods, and those using folkloric methods were recoded as ‘0’.

#### Independent variable

Sixteen independent variables were considered in this study based on their significant associations with modern contraceptive use in previous studies [14, 22–24]. These variables have been broadly grouped into individual-level variables and contextual-level variables. The individual-level variables were age (15–19, 20–24, 25–29, 30–34, 35–39, 40–44 and 45–49 years), marital status (never married, married, cohabiting, widowed and divorced), religion (Christianity, Islam, traditional and no religion), employment status (not working and working), age at first sex (less than 15 years, 15–19 years and 20 years and above), parity (no birth, one birth, two births, three births, four or more births), level of education (no education, primary, secondary and higher), exposure to newspaper (no and yes), exposure to radio (no and yes), and exposure to television (no and yes).

The contextual-level variables were place of residence (urban and rural), wealth index (poorest, poorer, middle, richer and richest), community-level literacy-proportion of women who can read and write (low, medium and high), community-level socio-economic status - the proportion of women in the richest household quintile (low, medium and high), community knowledge of modern contraceptive method (low, medium and high), and sub-region (West, East, Central and Southern). The countries in West Africa were Burkina Faso, Benin, Cote d’Ivoire, Ghana, Gambia, Guinea, Mali, Nigeria, Sierra Leone, Senegal, and Togo. Burundi, Cameroon, Ethiopia, Gabon, Kenya, Comoros, Malawi, Rwanda, Uganda, Zambia, and Zimbabwe were in East Africa. Angola, Congo DR, Congo, Liberia, and Chad were in Central Africa whiles Lesotho, and Namibia were in Southern Africa [25].

### Statistical analyses

The data were analysed with Stata version 16.0. The analysis was conducted in three steps. The first step was the graphical representation of the prevalence of modern contraceptive use among women with no fertility intention in SSA. The second step was a bivariate analysis that calculated the proportion of modern contraceptive use across the explanatory variables with their *p*-values derived from a chi-square test of independence. To check for high correlation among the explanatory variables, a test for multicollinearity was carried out using the variance inflation factor (VIF) and the results showed no evidence of high collinearity (Mean VIF = 1.44, Maximum VIF = 2.15, and Minimum VIF = 1.04). All the variables that showed statistical significance from the Table 2 were moved to the third step of the analysis. In the third step of the analysis, a multilevel logistic regression analysis comprising fixed effects and random effects was fitted. The results of the fixed effects of the model were presented as adjusted odds ratio (aOR) while the random effects were assessed with Intra-Cluster Correlation (ICC). Model comparison was done using the log-likelihood ratio (LLR) and Akaike’s Information Criterion (AIC) tests. Four models were fitted in examining the individualand contextual-level factors associated with  modern contraceptive use. The four models comprised an empty model (Model 0) which shows the variations in the use of modern contraceptives in the absence of any explanatory variable. Model I adjusted for the individual-level variables, Model II adjusted for the contextual-level variables and Model III adjusted for all the explanatory variables. Results were presented as adjusted odds ratios (aOR) at 95% Confidence Interval. All frequency distributions were weighted (v005/1000000) while the survey command (SVY) in Stata was used to adjust for the complex sampling structure of the data in the regression analyses.

### Ethical approval

The DHS reports that the DHS surveys have been reviewed and approved by Inner City Fund (ICF) Institutional Review Board (IRB) as well as Ethics Boards of partner organisations of the various countries such as the Ministries of Health. The DHS follows the standards for ensuring the protection of respondents’ privacy. ICF International ensures that the survey complies with the U.S. Department of Health and Human Services’ regulations for the respect of human subjects. This was a secondary analysis of data and therefore no further approval was required since the data is available in the public domain. Further information about the DHS data usage and ethical standards are available at http://goo.gl/ny8T6X (Table 1).

**Table 1:** Description of the study sample

Country	Year of survey	Women aged 15–49 years	Women with no fertlity intention who had information on contraceptive use	Women with complete cases
Angola	2015–16	14,379	339	339
Burkina Faso	2010	17,087	3419	3417
Benin	2018	15,928	3178	3176
Burundi	2016–17	17,269	5790	5787
Congo DR	2013–14	18,827	3368	3366
Congo	2011–12	10,819	1458	1457
Cote D’Ivorie	2011–12	10,060	1365	1364
Cameroon	2018	15,426	2928	2926
Ethiopia	2016	15,683	4718	4715
Gabon	2012	8422	1392	1391
Ghana	2014	9396	2264	2263
Gambia	2013	10,233	1071	1071
Guinea	2012	10,874	1485	1484
Kenya	2014	31,079	5318	5314
Comoros	2012	5329	620	620
Liberia	2013	9239	2001	2000
Lesotho	2014	6621	3439	1716
Mali	2018	10,424	1715	1714
Malawi	2015–16	24,562	8237	8232
Nigeria	2018	41,821	6931	6926
Namibia	2013	10,018	2788	2786
Rwanda	2014–15	13,497	4644	4641
Sierra Leone	2013	16,658	1663	1662
Senegal	2010–11	15,688	2266	2265
Chad	2014–15	17,719	1753	1752
Togo	2013–14	9480	2220	2219
Uganda	2016	18,506	5483	5479
Zambia	2018	13,683	4165	3978
Zimbabwe	2015	9955	3496	3494
**Total**			89,517	87,554

## Results

### Prevalence of modern contraceptive use among women with no fertility intention in sub-Saharan Africa

**Fig. 1:**
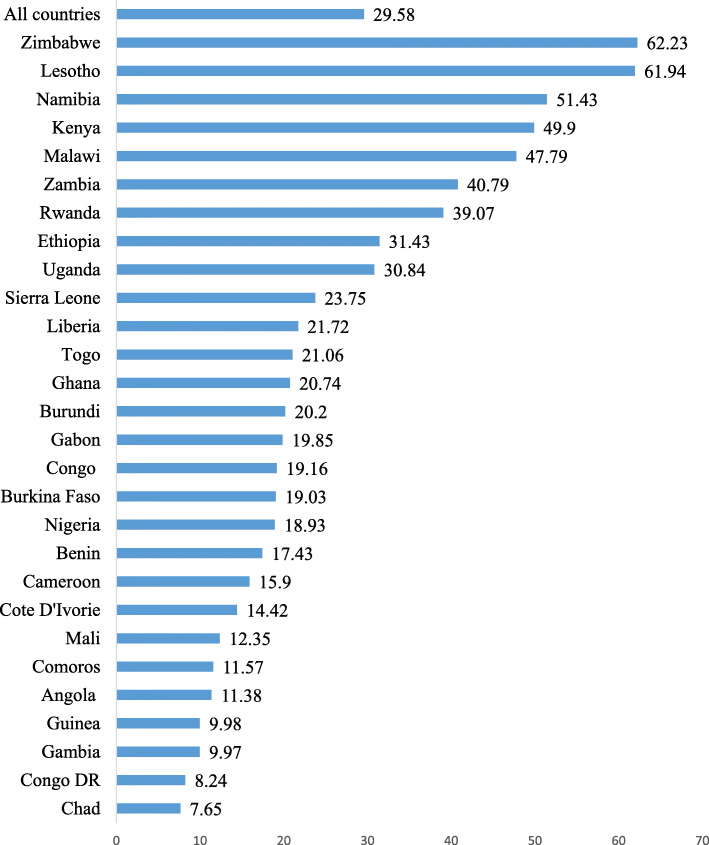
Prevalence of modern contraceptive use among women with no fertility intention in sub-Saharan Africa

Figure 1 shows the prevalence of modern contraceptive use among women with no fertility intention in 29 sub-Saharan African countries. The overall prevalence of modern contraceptive use among women with no fertility intention in the 29 sub-Saharan African countries considered in this study was 29.6%. In terms of country-based analysis, Zimbabwe (62.2%) had the highest prevalence of modern contraceptive use while Chad (7.7%) had the lowest prevalence.

### Distribution of modern contraceptive use by individual- and contextual-level variables

Table 2 shows the distribution of modern contraceptive use among women with no fertility intention by individual and contextual variables. With the individual-level factors, the highest prevalence of modern contraceptive use was found among women aged 25–29 (38.2%), married women (32.2%), Christians (33.9%), working women (29.7%), those who had their first sex when they were 15–19 years (31.1%), women with two births (38.6%), those with higher level of education (41.8%), and women with exposure to newspaper (41.8%), radio (32.7%), and television (33%). In terms of the contextual-level factors, the highest prevalence of modern contraceptive use was found among women in urban areas (32.4%), those in the richest wealth quintile (36.5%), women in communities with medium community literacy levels (35.9%), and socio-economic status (36.0%), those in communities with low knowledge of modern contraceptives (30.9%), and those who lived in Southern Africa (55.4%). The chi-square test results showed statistically significant associations between all the individual and contextual variables and modern contraceptive use.

**Table 2:** Distribution of modern contraceptive use by individual and contextual-level variables

Variables	Weighted N	Weighted %	Modern contraceptive	p-values
**Age**				< 0.001
15–19	2164	2.5	15.6	
20–24	4600	5.3	35.0	
25–29	9619	11.0	38.2	
30–34	15,430	17.6	37.9	
35–39	19,635	22.4	34.4	
40–44	19,234	22.0	27.0	
45–49	16,852	19.3	14.8	
**Marital status**				< 0.001
Never married	5209	6.0	27.4	
Married	57,118	65.2	32.2	
Cohabiting	10,331	11.8	29.4	
Widowed	3607	7.2	13.4	
Divorced	8590	9.8	25.4	
**Religion**				< 0.001
Christianity	64,891	74.1	33.9	
Islam	19,161	21.9	16.9	
Traditional	1914	2.2	15.0	
No religion	1588	1.8	23.7	
**Employment status**				0.004
Not working	17,988	20.5	29.0	
Working	69,566	79.5	29.7	
**Age at first sex**				< 0.001
Less than 15	18,149	20.7	23.9	
15–19 years	54,679	62.5	31.1	
20 years and above	14,726	16.8	31.0	
**Parity**				< 0.001
No birth	1933	2.2	8.1	
One birth	3867	4.4	25.5	
Two births	8502	9.7	38.6	
Three births	12,988	14.8	36.4	
Four or more births	60,264	68.8	27.8	
**Level of education**				< 0.001
No education	30,977	35.4	17.6	
Primary	92,933	37.6	34.7	
Secondary	20,324	23.2	37.5	
Higher	3302	3.8	41.8	
**Exposure to newspaper**				< 0.001
No	71,687	81.9	26.9	
Yes	15,867	18.1	41.8	
**Exposure to radio**				< 0.001
No	34,741	39.7	24.9	
Yes	51,813	60.3	32.7	
**Exposure to television**				< 0.001
No	54,414	62.1	27.5	
Yes	33,140	37.9	33.0	
**Residence**				< 0.001
Urban	30,434	34.8	32.4	
Rural	57,120	65.2	28.1	
**Wealth index**				
Poorest	16,062	18.4	22.1	< 0.001
Poorer	17,170	19.6	26.7	
Middle	17,582	20.1	29.4	
Richer	18,341	20.9	32.1	
Richest	18,399	21.0	36.5	
**Community literacy level**				< 0.001
Low	33,855	38.7	24.5	
Medium	29,145	33.4	35.9	
High	24,554	28.0	29.1	
**Community socioeconomic status**				< 0.001
Low	47,293	54.0	25.4	
Medium	10,087	11.5	36.0	
High	30,174	34.5	34.0	
**Community knowledge of modern contraceptive method**				< 0.001
Low	81,646	93.3	30.9	
Medium	5908	6.7	11.9	
**Sub-region**				< 0.001
West Africa	27,561	31.5	17.7	
East Africa	46,577	53.2	37.3	
Central Africa	8914	10.2	13.1	
Southern Africa	4502	5.1	55.4	

NB: p-values obtained from chi-square test

### Factors associated with modern contraceptive use among women with no fertility intention in sub-Saharan Africa

#### Measures of association (fixed effects)

Table 3 shows the results of the multilevel logistic regression analyses on the association between modern contraceptive use among women with no fertility intention and the individual- and contextual-level variables. With the individual-level factors, women aged 45–49 had lower odds of using modern contraceptive (aOR = 0.33, 95% CI = 0.28, 0.39) compared to those aged 15–19. Compared to married women, women who were never married, cohabiting, widowed or divorced were less likely to use modern contraceptives. The odds of modern contraceptive use decreased among Muslims, Traditionalists, and women with no religion compared to Christians. Women who were not working were less likely to use modern contraceptives compared to those who were working (aOR = 0.89, 95% CI = 0.85, 0.93). Compared to women who had their first sex at the age below  15, those who had their first sex at age 15–19 were more likely to use modern contraceptives (aOR = 1.12, 95% CI = 1.07,1.17). Women with four or more births were more likely to use modern contraceptives compared to those with no birth (aOR = 8.79, 95% CI = 7.13, 10.82). The likelihood of modern contraceptive use also increased with the level of education with the highest likelihood among those with higher education compared to those with no formal education (aOR = 1.93, 95% CI = 1.75, 2.13). Women who were exposed to newspaper (aOR = 1.15, 95% CI = 1.10, 1.20) and radio (aOR = 1.21, 95% CI = 1.17, 1.26) had higher odds of modern contraceptive use compared to those who were not.

**Table 3:** Mixed-effects results on the predictors of modern contraceptive use among women with no fertility intention in sub-Saharan Africa

Variables	Model 0	Model I aOR(95%CI)	Model II aOR(95%CI)	Model III aOR(95%CI)
**Age**				
15–19		1		1
20–24		1.20^*^ (1.04–1.40)		1.03 (0.88–1.20)
25–29		1.26^**^ (1.08–1.46)		0.95 (0.81–1.17)
30–34		1.26^**^ (1.08–1.47)		0.92 (0.78–1.08)
35–39		1.13 (0.74–1.32)		0.86 (0.73–1.01)
40–44		0.86 (0.74–1.01)		0.66^***^ (0.56–0.78)
45–49		0.43^***^ (0.37–0.50)		0.33^***^ (0.28–0.39)
**Marital status**				
Married		1		1
Not married		0.72^***^ (0.66–0.77)		0.59^***^ (0.55–0.65)
Cohabiting		0.61^***^ (0.58–0.64)		0.73^***^ (0.69–0.76)
Widowed		0.34^***^ (0.31–0.37)		0.31^***^ (0.28–0.33)
Divorced		0.56^***^ (0.53–0.60)		0.55^***^ (0.52–0.59)
**Religion**				
Christianity		1		1
Islam		0.53^***^ (0.50–0.55)		0.60^***^ (0.57–0.63)
Traditional		0.51^***^ (0.44–0.57)		0.78^***^ (0.69–0.89)
No religion		0.72^***^ (0.64–0.81)		0.81^***^ (0.75–0.91)
**Employment status**				
Working		1		1
Not working		1.00 (0.96–1.04)		0.89^***^ (0.85–0.93)
**Age at first sex**				
Less than 15		1		1
15–19		1.23^***^ (1.18–1.28)		1.12^***^ (1.07–1.17)
20+		1.15^***^ (1.09–1.22)		0.96 (0.91–1.02)
**Parity**				
No birth		1		1
One birth		3.41^***^ (2.81–4.14)		3.66^***^ (3.00–4.48)
Two births		6.13^***^ (5.03–7.47)		7.59^***^ (6.18–9.32)
Three births		6.30^***^ (5.16–7.70)		8.85^***^ (7.19–10.90)
Four or more births		5.55^***^ (4.54–6.77)		8.78^***^ (7.13–10.82)
**Education**				
No education		1		1
Primary		1.96^***^ (1.88–2.05)		1.56^***^ (1.49–1.63)
Secondary		2.05^***^ (1.95–2.16)		1.75^***^ (1.65–1.85)
Higher		2.23^***^ (2.03–2.45)		1.93^***^ (1.75–2.13)
**Exposure to newspaper**				
No		1		1
Yes		1.40^***^ (1.34–1.46)		1.15^***^ (1.10–1.20)
**Exposure to radio**				
No		1		1
Yes		1.31^***^ (1.26–1.35)		1.21^***^ (1.17–1.26)
**Exposure to television**				
No		1		1
Yes		0.97 (0.93–1.01)		0.98 (0.94–1.02)
**Residence**				
Rural			1	1
Urban			1.05^*^ (1.01–1.10)	1.06^**^ (1.02–1.11)
**Wealth index**				
Poorest			1	1
Poorer			1.36^***^ (1.39–1.43)	1.26^***^ (1.19–1.33)
Middle			1.53^***^ (1.45–1.61)	1.34^***^ (1.27–1.41)
Richer			1.70^***^ (1.61–1.80)	1.44^***^ (1.35–1.52)
Richest			1.99^***^ (1.86–2.13)	1.55^***^ (1.43–1.67)
**Community literacy level**				
Low			1	1
Medium			1.27^***^ (1.23–1.32)	1.11^***^ (1.06–1.16)
High			1.12^***^ (1.07–1.17)	0.95 (0.90–1.00)
**Community socioeconomic status**				
Low			1	1
Medium			1.14^***^ (1.08–1.20)	1.17^***^ (1.10–1.23)
High			1.06^*^ (1.01–1.12)	1.13^***^ (1.07–1.19)
**Community knowledge of modern contraceptive method**				
Low			1	1
Medium			0.77^***^ (0.70–0.84)	0.81^***^ (0.74–0.89)
**Sub-region**				
West Africa			1	1
East Africa			2.62^***^ (2.52–2.72)	2.16^***^ (2.07–2.27)
Central Africa			0.66^***^ (0.62–0.72)	0.57^***^ (0.52–0.62)
Southern Africa			5.08^***^ (5.08–5.85)	5.29^***^ (4.86–5.76)
**Random effect result**				
PSU variance (95% CI)	0.03 (0.02–0.04)	0.02 (0.02–0.03)	0.02 (0.02–0.03)	0.02 (0.01–0.03)
ICC	0.009398	0.0073918	0.0066778	0.0059027
LR Test	Chi-square = 93.90, *p* < 0.0001	Chi-square = 60.28, p < 0.0001	Chi-square = 52.94, p < 0.0001	Chi-square = 39.24, p < 0.0001
Wald chi-square	Reference	8014.01^***^	6928.45^***^	11,041.57^***^
Model fitness				
Log-likelihood	−52,079.447	−47,165.892	−48.68.293	−44,895.43
AIC	104,162.9	94,387.78	96,166.59	89,872.86
N	87,554	87,554	87,554	87,554
Number of clusters	1605	1605	1605	1605

Exponentiated coefficients; 95% confidence intervals in brackets

^*^ *p* < 0.05, ^**^ *p* < 0.01, ^***^ *p* < 0.001,

1 = Reference category; ICC = Intra-Class Correlation; AIC = Akaike’s Information Criterion;

Model 0 = The null model, a baseline model without any determinant variable

Model I = Individual-level variables

Model II = Contextual-level variables

Model III = The final model adjusted for individual- and contextual-level variables

In terms of contextual factors, women in communities with medium knowledge of modern contraceptive method (aOR = 0.81, 95% CI = 0.75, 0.89) were less likely to use modern contraceptives. Conversely, women in urban areas (aOR = 1.06, 95% CI = 1.02, 1.11), women with richest wealth quintile (aOR = 1.55, 95% CI = 1.43,1.67), those in communities with medium literacy level (aOR = 1.11, 95% CI = 1.06, 1.16) and medium community socio-economic status (aOR = 1.17, 95% CI = 1.10, 1.23) had higher odds of modern contraceptive use. Across the geographic regions in SSA, Southern Africa had higher odds of modern contraceptive use (aOR = 5.29, 95% CI = 4.86, 5.76) compared to those in West Africa.

#### Measures of variation (random effects)

The results of the random effects on the association between modern contraceptive use among women with no fertility intention and the individual and contextual variables show variations in all the models. In Model 0, the clustering of the primary sampling units (PSUs) accounted for significant variations in the odds of modern contraceptive use (σ2 = 0.03, 95% CI 0.02–0.04). Model 0 showed that 0.9% of the total variation in modern contraceptive use was attributed to the variance between clusters (ICC = 0.0093). The between-cluster variance was slightly smaller (ICC = 0.0079) in Model I (with individual-level factors only). From Model I, the ICC decreased in Model II (contextual level only model) (ICC = 0.0072). It then increased in Model III (ICC = 0.0067) where all the independent variables (both individual and contextual variables) were considered. This indicates that differences in the clustering of the PSUs account for the variations in modern contraceptive use. The highest log- likelihood (− 44,895.43) and the lowest AIC (89,872.86) were used to determine the best fit model (Table 3).

## Discussion

In this study, we investigated the prevalence and correlates of modern contraceptive use among women with no fertility intention in SSA. We found the overall prevalence of modern contraceptive use among the selected women to be 29.6%. This low prevalence suggests that the use of modern contraceptives for fertility prevention is still a problem among women in SSA. In terms of country-based analysis, Zimbabwe (62.2%) had the highest prevalence of modern contraceptive use followed by Lesotho (7.7%). Chad (7.7%) had the lowest prevalence of modern contraceptive use. This finding agrees with that of Yaya et al. (2018), who reported low contraceptive use in several sub-Saharan African countries, including Chad. However, Chikandiwa et al. [26] reported higher use of modern contraception among Zimbabwean women than Kenyan women. In explaining this finding, a study in Zimbabwe showed that over the years, the post-independence Zimbabwean government had encouraged women to use contraceptives, hence resulting in high prevalance of contraceptives among Zimbabwean women  [27]. Women aged 45–49 had lower odds of using modern contraceptives compared to those aged 15–19. This finding resonates with that of Adebowale et al. [28]. The lower contraceptive use might be because women aged 15–19 are still in school and as a result might not want to have children to interrupt their academic activities [28]. Another reason could be that younger women (age 15–19) are highly sexually active and had to use modern contraceptives to prevent unwanted pregnancies [29]. Relatedly, women who were never married, cohabiting women, widows, and divorcees were less likely to use modern contraceptives compared to married women. The reason could be that women with no births and those with few births were sexually active and fertile, which demanded that they used more contraceptives to prevent pregnancies. On the other hand, widowed women and divorcees might not be in any sexual relationship, therefore resulting in their low use of modern contraceptives.

With mass media exposure, women who were exposed to newspaper and radio had higher odds of modern contraceptive use, compared to those who were not. This finding highlights the role of mass media in spreading information regarding family planning. Previous studies in Ethiopia [30] and Nigeria [31], as well as Burkina Faso and Senegal [32] have reported the effectiveness of mass media messages in promoting contraceptive use. Ownership of a radio and exposure to family planning radio messages independently encourage women to use modern methods of contraception [33]. This findings therefore suggest the need for sub-Saharan African countries to use mass media as a channel to spread information regarding the use of modern contraceptives to women. This has a capacity to increase contraceptive use among women with no fertility intention.

The odds of modern contraceptive use decreased among Muslims, Traditionalists and women with no religion compared to Christians. A negative relationship between Muslim women and family planning use has been reported by previous studies [34, 35]. Our finding agrees with previous studies conducted in Ethiopia [36, 37], Nigeria [28, 34, 35] and Ghana [38]. The reason for this might be that religious resistance for contraceptive uptake may be more pronounced in Islam than other religions [7, 39]. A study by Hani et al. [40] revealed a strong opposition by husband to the use of contraceptive. This was found as a reason for non-use of contraceptives among Muslim women. As Hani et al. [40] suggested, participation of husband in counseling and involvement of religious leaders in decisions on contraceptive use could improve its use among Muslim women.

Consistent with previous studies in Ethiopia [37], Malawi [41], and Ghana [42], this study recorded lower odds of modern contraceptives use among women who were not working. In most sub-Saharan African countries, contraceptive access is not completely free. At health facilities, levies are charged. Apart from this, distance from family planning clinics is considered as far for most women, particularly those from rural communities. Even in urban areas, family planning clinics are not evenly situated. Transportation cost becomes a barrier to utilization of family planning services [28]. Women need some level of financial autonomy to be able to purchase and use modern contraceptives. Whereas working women may be able to cater for the cost involved in using modern contraceptives, their counterparts who are not working may not [37, 41, 42]. Women who are engaged in occupations that disallow frequent maternity leaves might cause them to use contraceptives.

Moreover, we recorded lower odds of modern contraceptives use among women living in rural areas, and those with medium community knowledge of modern contraceptive method. Similarly, Adebowale et al. [28] reported lower use of modern contraceptives among women with no fertility intention in rural areas in Nigeria. As noted by Asresie et al. [7], inadequate access to family planning service is one of the predominant reasons for the non-use of contraceptives. Thus, the lower use of modern contraceptives among inhabitants of rural areas may be attributed to poor access or unavailability of health facilities in rural areas. Other reasons for low use of modern contraceptives in rural areas could be poor spousal communication, sociocultural norms (especially the husband’s role as the primary decision-maker), fear of side-effects and a lack of knowledge [43]. This finding highlights the need for development of programs to increase contraceptive use among women with no fertility intention while taking into consideration the rural-urban disparities.

Finally, we found level of education and community literacy to be significantly associated with the use of modern contraceptives among the study participants. As Nyarko [42] emphasized, well-educated people have contraceptive advantages in two ways. First, their level of education can provide them with accurate knowledge about contraception, contraceptive methods and their benefits. Second, their period of education may encourage them to use modern contraceptive methods to avoid getting pregnant while in school. In terms of community literacy, our study agrees with the findings of Ahinkorah [22], who reported low use of modern contraceptives use among women living in communities with low literacy in SSA. Our study also showed that women in the richest wealth quintile and those in communities with middle socioeconomic status recorded higher odds of modern contraceptives utilization. The use of contraceptives comes with its associated financial burden. While richest women may be in the position to skip any financial hurdles to the use of modern contraceptives, poor women may not [21, 22].

### Strengths and limitations

The main strength of this study lies in the use of nationally representative data of each of the countries represented in the study. With this, the findings are generalizable to all women in the countries studied. Another strength lies in the use of sophisticated data collection methods, with experienced field assistants, which generated a higher response rate. We also employed higher order statistical tools for the analysis, which ensured rigorous analysis of the data. Despite these strengths, the study has some limitations that need to be acknowledged. First, with the cross-sectional research approach adopted, we cannot make causal inferences among the studied variables. Also, given the retrospective nature of reporting that characterizes demographic data, the data are likely to be subjected to recall biases. Relatedly, issues of social desirability bias may also be present.

## Conclusion

There is a relatively low prevalence of modern contraceptive use among women with no fertility intention in sub-Saharan Africa, with cross-country variations. Women’s age, age at first sex, level of education, mass media exposure, religion, employment status, place of residence, community literacy level and community socio-economic status were found to be associated with modern contraceptive use.. It is, therefore, important for policy makers policies to consider these factors when designing and implementing programmes or policies take to increase contraceptive use among women who have no intention to give birth. Also, policymakers and other key stakeholders should intensify mass education programmes to address disparities in modern contraceptive use among women. Such education programmes should target both rural and urban communities to increase women's knowledge and use of modern contraceptives.

## Data Availability

The dataset is freely available for download at: https://dhsprogram.com/data/available-datasets.cfm.

## Abbreviations

aOR: Adjusted Odds Ratio
CI: Confidence Interval
GDHS: Ghana Demographic and Health Survey
DHS: Demographic and Health Survey
PHC: Population and Housing Census
NHIS: National Health Insurance Scheme
